# Identifying top ten predictors of type 2 diabetes through machine learning analysis of UK Biobank data

**DOI:** 10.1038/s41598-024-52023-5

**Published:** 2024-01-24

**Authors:** Moa Lugner, Araz Rawshani, Edvin Helleryd, Björn Eliasson

**Affiliations:** 1 Institute of Medicine, Sahlgrenska Academy, University of Gothenburg, Gothenburg, Sweden; 2 Department of Medicine, Sahlgrenska University Hospital, Gothenburg, Sweden

**Keywords:** Machine learning, Endocrine system and metabolic diseases, Type 2 diabetes

## Abstract

The study aimed to identify the most predictive factors for the development of type 2 diabetes. Using an XGboost classification model, we projected type 2 diabetes incidence over a 10-year horizon. We deliberately minimized the selection of baseline factors to fully exploit the rich dataset from the UK Biobank. The predictive value of features was assessed using shap values, with model performance evaluated via Receiver Operating Characteristic Area Under the Curve, sensitivity, and specificity. Data from the UK Biobank, encompassing a vast population with comprehensive demographic and health data, was employed. The study enrolled 450,000 participants aged 40–69, excluding those with pre-existing diabetes. Among 448,277 participants, 12,148 developed type 2 diabetes within a decade. HbA1c emerged as the foremost predictor, followed by BMI, waist circumference, blood glucose, family history of diabetes, gamma-glutamyl transferase, waist-hip ratio, HDL cholesterol, age, and urate. Our XGboost model achieved a Receiver Operating Characteristic Area Under the Curve of 0.9 for 10-year type 2 diabetes prediction, with a reduced 10-feature model achieving 0.88. Easily measurable biological factors surpassed traditional risk factors like diet, physical activity, and socioeconomic status in predicting type 2 diabetes. Furthermore, high prediction accuracy could be maintained using just the top 10 biological factors, with additional ones offering marginal improvements. These findings underscore the significance of biological markers in type 2 diabetes prediction.

## Introduction

Type 2 diabetes has emerged as one of the most prevalent chronic diseases worldwide, posing a significant economic and health burden on both individuals and society^1^. However, to some extent it may be preventable or delayed through lifestyle interventions and pharmacotherapy. Previous studies have demonstrated the efficacy of such measures in preventing or postponing the onset of type 2 diabetes^2–5^.

Moreover, recent studies have revealed that nearly half of all adults affected by type 2 diabetes are neither diagnosed nor aware of their condition^1^. The asymptomatic nature of the initial stages of prediabetes and diabetes increases the likelihood of developing micro- and macrovascular complications prior to the initiation of glucose management interventions^6^. The identification of high-risk individuals even before the onset of prediabetes enables comprehensive follow-up to be initiated in this population, thereby ensuring timely diagnosis and glucose management.

Recent advances in machine learning and "big data" offer transformative potential in health research, allowing deeper insights from complex datasets that were previously elusive. However, when it comes to predicting type 2 diabetes, the field reveals a marked inconsistency, particularly in feature selection^7^. Most studies prioritize achieving high prediction accuracy, which often leads them to exclude features believed to be less impactful. It has been shown that a large proportion of prediction models are trained on fewer than 20 features^8^. In some instances, the specific features utilized are not even presented^8^. There is a lack a consensus regarding which features to include in prediction models for type 2 diabetes, and our study seeks to address this gap. The purpose of this study is to identify the most significant factors that predict subsequent type 2 diabetes. An agnostic approach with minimal preconceptions and human intervention will be employed to achieve this objective. The utilization of the vast amount of information collected in the UK Biobank, combined with cutting-edge machine learning technologies, offers the opportunity to unveil previously unidentified factors contributing to diabetes risk. This approach also allows us to elucidate the relationships between already established risk factors, and to determine which factors possess the highest predictive power for type 2 diabetes.

## Methods

### Data

All data utilized in this study were obtained from the UK Biobank, a comprehensive biomedical database that has accumulated extensive health information from 502,625 individuals resident in the UK. Between 2006 and 2010, baseline assessments were conducted on participants aged 40 to 69 years through a combination of touchscreen questionnaires, nurse-led interviews, and various tests and measurements. Information on dietary habits were assembled using a food frequency questionnaire, that has been shown to reliably rank participants according to intakes of the main food groups^9^. Participants' physical activity level was assessed using an adapted version of the international physical activity questionnaire (IPAQ)^10^. During the verbal interview, participants were queried about various aspects of their personal and medical history, including early life factors, employment status, medical conditions, medications, and past surgical procedures. The physical measurements carried out at baseline assessment included blood pressure, arterial stiffness, bone density, hand grip strength, hearing test, eye measures, and spirometry. Thirty blood assays, which were selected based on their established risk factors for disease or diagnostic measures, were collected, along with eight urine assays.

### Outcome

The primary endpoint of this study was the incidence of type 2 diabetes within a 10-year period following the baseline assessment (3650 days). To determine this outcome, we utilized the "first occurrence" category in the UK Biobank, which contains two data fields for each code mapped to 3-character ICD-10. The first field represents the date of the first reporting of the ICD code, and the second field contains the source where the code was initially recorded. The sources included Read code information in the Primary Care data, ICD-9 and ICD-10 codes in the Hospital inpatient data, ICD-10 codes in Death Register records, and self-reported medical condition codes reported at subsequent UK Biobank assessment centre visits. In this study, the outcome variable was defined as any report of ICD code E11 (non-insulin-dependent diabetes mellitus) during the 10-year study period.

### Exclusion criteria

Individuals who had diabetes at baseline were excluded from the study. Specifically, those who had either self-reported having diabetes at the baseline interview or had a measured HbA1c > 48 mmol/mol or had a diagnosis of diabetes recorded in their hospital or primary care records, regardless of the type of diabetes. However, women who had only had diabetes during pregnancy were not excluded. Additionally, participants who did not have a full 3650 days of follow-up due to either death or withdrawn consent were also excluded from the study.

### Variable selection

Our variable selection approach was both intentional and informed by expert judgment. From the outset, our primary objective was to preserve as much information as possible to facilitate an unbiased analysis rich in details about the participants. With this aim in mind, we began by excluding any variables that were collected after the baseline assessment, as we were committed to using only baseline data for predictive modeling. Subsequently, we meticulously assessed the baseline data to guarantee its relevance and accuracy. This assessment was a manual process where each variable was assessed for its contribution to a comprehensive understanding of a participant's health, lifestyle, and socio-economic status. Only variables that were deemed unrelated to these key areas, or that did not provide additional insight, were set aside. For instance, data attributes such as the serial numbers of measuring devices, the duration of tests, or reasons for skipping certain tests were disregarded since they did not impart meaningful information about the participants. In situations where duplicate variables existed, like two separate measurements of a participant's blood pressure, we averaged the values and represented them with a single variable. Following this, we identified 111 variables that had more than 70% missing observations and these were removed from the dataset. To improve the informative value of the dataset, certain variables that were missing in the UK Biobank were created using the available data. This included the creation of variables such as estimated glomerular filtration rate (eGFR), total weekly alcohol intake, pulse pressure and mean arterial pressure. Additionally, information about first degree relatives with type 2 diabetes was combined into a score ranging from 0 to 2 depending on the number of relatives with diabetes (2 indicating two or more relatives with diabetes). After this meticulous selection and refinement process, our dataset comprised 419 variables deemed most suitable for model development, and a comprehensive list of these variables can be found in a separate document as well as formulas for creating new variables (Supplements).

### Model development

The classification model used in this study employed the extreme gradient boosting (XGBoost) algorithm, which is a widely used ensemble learning technique^11^. XGBoost is known for its high predictive accuracy and computational efficiency, making it a popular choice for classification and regression tasks^12,13^. The first step in the analysis was to split the data into two sets, a training dataset, and a validation dataset. The training set contained 80% of the total observations, while the remaining 20% were allocated to the validation set. To ensure that the proportion of individuals with the outcome of interest was balanced in both sets, the split was stratified based on the outcome variable. Once the split was completed, the training dataset underwent additional preprocessing. All categorical features were converted into numerical variables using one-hot encoding. Variables with very low variance were identified and removed to enhance model stability. Notably, to address class imbalances in the training data, the majority class was downsampled to achieve a 1:3 ratio. This downsampling was only applied to the training set.

While certain preprocessing steps such as one-hot encoding and variance filtering were learned from the training data, their transformations were consistently applied to both the training and validation sets. However, the downsampling step was exclusively applied to the training data and did not affect the validation dataset.

### Hyperparameter tuning

Latin hypercube sampling is a method for generating sets of parameter values that are evenly distributed across the parameter space. This method can be used for hyperparameter tuning in machine learning to efficiently search for the optimal combination of hyperparameters^14^. The grid search algorithm involves defining a grid of hyperparameters to be tested, while the Latin hypercube sampling method randomly selects values for the hyperparameters within defined bounds. The five-fold cross-validation technique involves splitting the data into five subsets, training the model on four subsets, and testing on the fifth subset, and repeating this process five times. The average performance across all iterations is used as the evaluation metric. The hyperparameters tuned included the number of variables randomly sampled as candidates at each split (mtry), the number of trees (trees), the minimum node size (min_n), the depth of the tree (tree depth), the minimum loss reduction required to make a further partition on a leaf node (loss reduction), and the fraction of samples used to train each tree (sample size). The objective was to find the combination of hyperparameters that resulted in the highest receiver operating characteristic area under the curve (ROC-AUC).

### Model evaluation

Although the primary performance metric was ROC-AUC, due to its reliability in unbalanced datasets^13,14^, we also provided a comprehensive set of other metrics for transparency. These include accuracy, sensitivity, specificity, precision, F1-measure, PR-AUC, and confusion matrices. 95% confidence intervals were calculated for all performance metrics using bootstrapping with 1000 replications to quantify the uncertainty of the model evaluation. All evaluations were conducted on the validation dataset, which was excluded from model training.

### Shapley values (model interpretability and feature importance)

SHapley Additive explanation (Shap) is a technique used to explain the predictions made by machine learning models^15,16^. It originates from cooperative game theory and is based on the concept of Shapley values. The Shap value for a feature is the average marginal contribution of that feature to the model's prediction, after accounting for all possible combinations of features. To calculate the Shap value, the impact of a feature on the model's prediction is compared with and without the feature. This provides a measure of the feature's importance, considering its interactions with other features. The Shap technique can be used for both local interpretabilities, to understand individual predictions, and global interpretability, to identify drivers of predictions across the entire dataset. In this study, only global interpretability tools were used.

### Model with selected features

The main model contained 419 features, and the top 10 features with the highest prediction value were identified using Shap values. A reduced XGBoost model was developed using the same train/validation split, feature preprocessing, and hyperparameter tuning as the main model. The performance of both the main and reduced models was compared based on their ability to predict using various metrics such as ROC-AUC, accuracy, sensitivity, and specificity.

### Sex-specific models

To compare important predictors of diabetes for women and men separately, two additional models were constructed by dividing the total population by sex. Shap summary graphs were then used to describe the 10 most important predictors for each sex. The development of these models followed the same procedure as the main model, except for the addition of sex-specific factors that were not included in the main model. For the female cohort, 30 factors related to menstruation, pregnancy, childbirth, menopause, and use of hormone replacement therapy were included. For the male cohort, the added features included relative age of first facial hair, relative age voice broke, hair/balding pattern, and number of children fathered.

All data preparation and model engineering were performed using R with RStudio Workbench version 1.4.1717-3. Tidymodels framework was utilized to construct the models^17^.

### Ethics approval

The present study adheres to the ethical standards of the Swedish Ethical Review Authority, which approved the research methodology, confirming compliance with the pertinent ethical principles and guidelines. All procedures involving human participants were performed in accordance with the Declaration of Helsinki and relevant guidelines/regulations. The UK Biobank obtained written informed consent from all participants prior to their inclusion in the study, ensuring that all methods were conducted in accordance with the aforementioned ethical standards.

## Result

The study enrolled a total of 448,277 participants, of whom 43.9% were male. The median follow-up duration was 4440 days, or approximately 12.16 years, with an interquartile range of 502 days. During this period, 12,148 individuals developed type 2 diabetes. The individuals who developed diabetes were found to have, on average, a higher age at recruitment by 2.5 years, a higher body mass index (BMI) of 31.5 kg/m^2^ compared to 27.0 kg/m^2^, and a higher body fat percentage of 34.9% compared to 31.2%. With respect to lifestyle factors, the group of individuals who developed diabetes exhibited a higher frequency of smoking and consumption of processed foods, while their weekly alcohol intake was similar to the group who did not develop diabetes. Additionally, a higher proportion of individuals in the diabetes group had a non-white ethnic background. The mean systolic blood pressure was found to be notably elevated in the diabetes group, with a recorded value of 144 mmHg, compared to 137.3 mmHg in the group without diabetes (Table 1).

**Table 1:** Baseline characteristics of study population stratified by incidence of diabetes during study period.

	Overall	No diabetes	Diabetes
n	448,277	436,129	12,148
Male (%)	43.9	43.6	55.5
Age (mean (SD))	56.1 (8.1)	56.0 (8.1)	58.6 (7.5)
BMI (mean (SD))	27.2 (4.6)	27.0 (4.5)	31.5 (5.5)
Body fat percentage (mean (SD))	31.3 (8.5)	31.2 (8.5)	34.9 (8.4)
Currently smoking (%)	33.8	33.7	38.7
Weekly alcohol intake (n. of units/week)	14.8 (18.8)	14.8 (18.7)	14.3 (21.8)
Systolic blood pressure (mean (SD))	137.4 (18.6)	137.3 (18.6)	143.9 (18.5)
Diastolic blood pressure (mean (SD))	82.3 (10.1)	82.2 (10.1)	85.3 (10.4)
HbA1c (mean (SD))	35.0 (3.7)	34.8 (3.6)	40.1 (4.2)
LDL (mean (SD))	3.6 (0.9)	3.6 (0.8)	3.5 (0.9)
HDL (mean (SD))	1.5 (0.4)	1.5 (0.4)	1.2 (0.3)
Triglycerides (mean (SD))	1.7 (1.0)	1.7 (1.0)	2.4 (1.3)
Lipid lowering treatment (%)	16.3	15.8	34.1

For continuous variables, means and standard deviations are reported. For categorical variables, percentages are reported. Alcohol intake is presented in terms of the number of standard units consumed per week, with one unit defined as 10 ml or 8 g of pure alcohol according to NHS guidelines.

**Figure 1:**
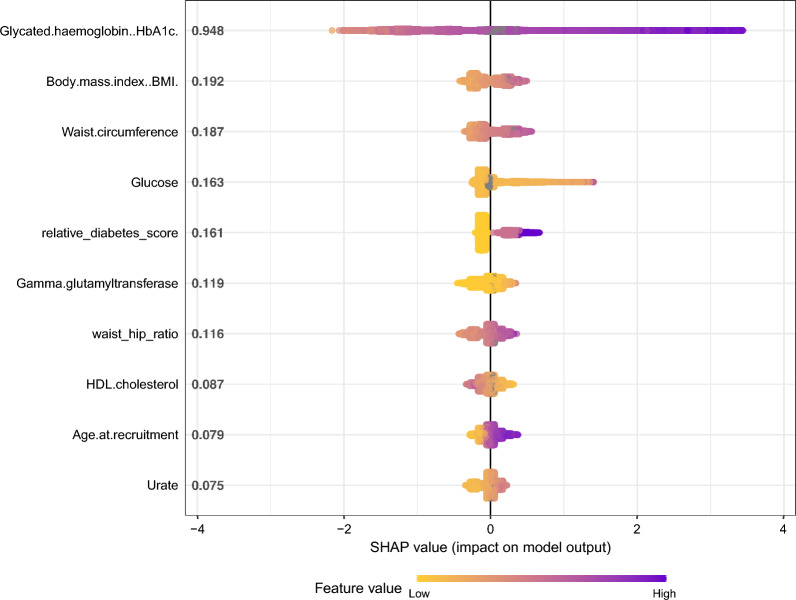
The Shap summary graph depicts the ranked mean absolute Shap values for each variable in the dataset on the y-axis. Each individual in the study is represented by a dot on the graph based on their respective Shap value. The points are stacked verticly where there’s a high density of shap values. The color of each dot corresponds to the raw variable value of that individual and feature, with purple indicating a high raw value and yellow indicating a low raw value.

HbA1c exhibited the strongest predictive power for type 2 diabetes, followed by BMI, waist circumference, blood glucose levels, the number of first-degree relatives with diabetes, GGT, waist-hip ratio, HDL cholesterol, age, and urate levels. Elevated raw variable values were positively associated with increased risk for diabetes across all variables except for HDL cholesterol, where a high value corresponded to a reduced diabetes risk (Fig. 1).

**Figure 2:**
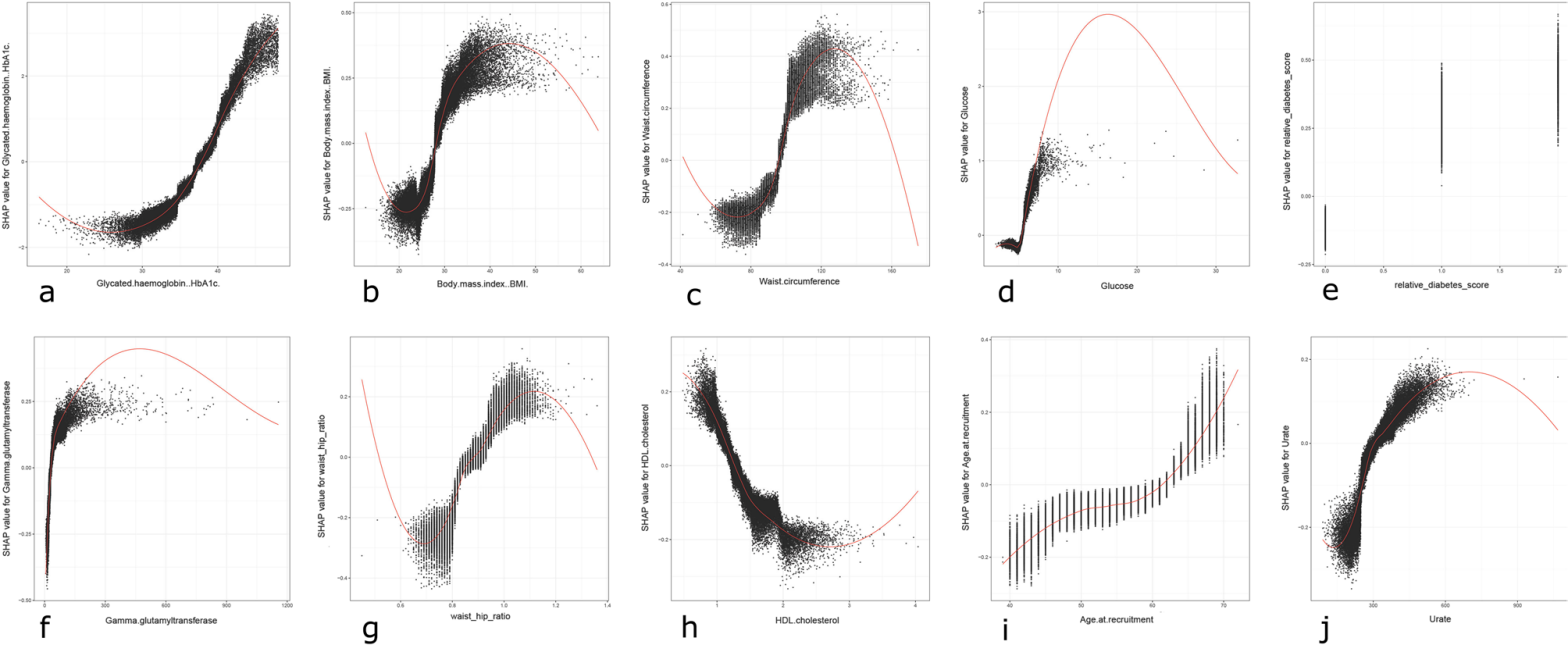
(**a**–**j**) The Shap dependency graphs are presented as scatterplots, with each participant being represented by a data point. These scatterplots depict the Shap value plotted against the underlying raw value for the variables under consideration. Shap values exceeding the y = 0 line are indicative of a higher risk of developing diabetes, while those falling below the line are associated with a lower risk. Due to interactions with other variables, the same raw variable value can generate different Shap values. As an illustration, the age-graph displays a wide range of Shap values for the same number of years, thus indicating the impact of other variables.

The dependency plots offer a deeper understanding of the association between a given variable and the risk of developing diabetes. In the case of HbA1c, the graph reveals that individuals with HbA1c levels < 38 mmol/mol are less prone to developing diabetes than those with HbA1c levels > 38 mmol/mol (as denoted by the point on the x-axis at y = 0). Moreover, the risk of developing type 2 diabetes increases almost linearly after the HbA1c values exceed 30 mmol/mol. In the case of BMI, this threshold is approximately 28 kg/m^2^. The risk appears to be low and similar for values between 18 and 25; however, it starts to rise sharply at 25 kg/m^2^. The curve reaches a plateau at around 40 kg/m^2^, beyond which all values appear to confer almost the same risk. The absence of any first-degree relatives with diabetes is associated with negative Shap values, while having two or more first-degree relatives with diabetes is linked to the highest risk of developing diabetes. Age appears to have a linear relationship with diabetes risk, with the risk increasing proportionally with the number of years. In the case of serum urate, the threshold appears to be located at 300 µMol/L, with values above this level being associated with a higher risk of diabetes. (Fig. 2).

Based on the analysis, the top five predictors for males were found to be HbA1c, plasma glucose, BMI, family history of diabetes, and GGT. For females, the most significant predictors were HbA1c, waist circumference, plasma glucose, family history of diabetes, and serum urate (Supplements).

### Model comparison

The hyperparameters selected for the main model were based on ROC-AUC and consisted of mtry = 273, trees = 1306, min_n = 32, tree depth = 11, loss reduction = 0.0006507575, and sample size = 0.6880322. The reduced model utilized final hyperparameters of mtry = 2, trees = 1931, min_n = 39, tree depth = 8, loss reduction = 10.91671, and sample size = 0.5617388. When applying the main model to predict the outcome on the validation set, it was able to detect 1554 individuals with diabetes, but missed 941 individuals. In comparison, the reduced model detected 1419 individuals with diabetes, but missed 1076. The main model accurately identified 81,259 individuals who did not develop diabetes, while the reduced model identified 81,185 individuals (Table 2).

**Table 2:** Main model: XGBoost model with all available features included.

Confusion matrix for main model				Confusion matrix for reduced model			
		Truth				Truth	
		1	0			1	0
Prediction	1	1554	5902	Prediction	1	1419	5976
	0	941	81,259		0	1076	81,185

Reduced model: XGBoost model with only the 10 most influential features based on Shap values. These matrices summarize the performance of each model in classifying instances as positive (1) or negative (0).

ROC-AUC for the main model on the validation set was 0.90 and ROC-AUC for the reduced model was 0.88. The accuracy of both models was 0.92. The sensitivity and specificity of the main model were 0.62 and 0.93, respectively, while the reduced model had a sensitivity of 0.57 and a specificity of 0.93 (Table 3).

**Table 3:** ROC-AUC: Plots the true positive rate against the false positive rate. AUC represents the area under this curve.

Model performance		
Metric	Main model	Reduced model
ROC-AUC	0.903 (95% CI 0.900–0.909)	0.881 (95% CI 0.875–0.888)
Accuracy	0.924 (95% CI 0.922–0.925)	0.921 (95% CI 0.920–0.923)
Sensitivity	0.623 (95% CI 0.603–0.641)	0.569 (95% CI 0.549–0.587)
Specificity	0.932 (95% CI 0.930–0.934)	0.931 (95% CI 0.930–0.933)
F1-measure	0.311 (95% CI 0.300–0.323)	0.287 (95% CI 0.275–0.300)
Precision	0.207 (95% CI 0.198–0.217)	0.192 (95% CI 0.183–0.201)
PR-AUC	0.291 (95% CI 0.275–0.309)	0.255(95% CI 0.239–0.272)

Accuracy: (TP + TN)/(TP + TN + FP + FN).

Sensitivity (or Recall): TP/(TP + FN).

Specificity: TN/(TN + FP).

F1-measure: Harmonic mean of precision and sensitivity.

Precision: TP/(TP + FP).

PR-AUC: Area under the precision-recall curve, plotting precision against recall.

TP = True Positive; TN = True Negative; FP = False Positive; FN = False Negative.

## Discussion

In this research study, our analysis revealed that HbA1c levels measured at baseline were the most influential factor for predicting the risk of developing type 2 diabetes within a 10-year timeframe. BMI, waist circumference, plasma glucose, family history of diabetes, GGT, waist-hip ratio, HDL cholesterol, age, and serum urate levels also exhibited significant predictive power. By utilizing these 10 readily available and cost-effective variables, we were able to predict the risk of diabetes with high precision.

Machine learning techniques have been previously shown to accurately predict the risk of future diabetes and other chronic diseases. The objective of our study was not to establish the feasibility of such predictions but rather to identify the most important factors that influence the risk of developing diabetes. The UK Biobank offers the advantage of detailed baseline information on participants, including lifestyle habits, body composition, and socio-economic background. Our results demonstrate that biological factors are the most significant predictors of diabetes risk, whereas information on lifestyle habits, food preferences, socio-economic status, and physical activity only have a minor impact on prediction accuracy in the UK Biobank cohort.

The metabolic syndrome is usually defined as a pathologic condition characterized by abdominal obesity, insulin resistance, hypertension, and hyperlipidemia^18^. It is in other words a presence of multiple metabolic risk factors for cardiovascular disease and diabetes^18^. Several of the strongest predictors in our study has been associated with this condition. The crucial role of HbA1c and plasma glucose in diabetes prediction models stem from their ability to serve as reliable markers of impaired glucose metabolism, thereby elevating the risk of diabetes development. Impaired fasting glucose (IFG) denotes serum glucose levels that fall below the diagnostic criteria for diabetes (7 mmol/L) but are above normal values, and individuals with such intermediate values have been shown to exhibit an elevated risk of developing type 2 diabetes (20). In our study, the cut-off for plasma glucose was just above 5 mmol/l, where higher values were associated with higher risk of subsequent type 2 diabetes. The cut-off for HbA1c was identified at 38 mmol/mol in our study. Previous research has shown that individuals with HbA1c levels between 39 and 46 mmol/mol are at high risk of developing type 2 diabetes, as stated by the American Diabetes Association (20). Our findings on both glucose and HbA1c levels are consistent with prior research, albeit indicating that the threshold for increased risk may be slightly lower.

GGT is an enzyme commonly used in clinical settings as a marker for liver function and alcohol consumption. However, emerging evidence suggests a significant and positive dose–response association between GGT levels and the incidence of type 2 diabetes^19^. Urate is known to be associated with both metabolic syndrome and diabetes. Elevated urate levels have been shown to precede the onset of both conditions, indicating that urate may be strongly linked to diabetes development^20^. Additionally, studies have demonstrated that insulin resistance can be improved by lowering uric acid levels in vitro, further supporting the potential role of urate in diabetes development^21^.

Three of the ten most influential predictors in our study were anthropometric measurements, specifically body mass index (BMI), waist circumference, and waist-to-hip ratio. Although BMI is commonly used, it provides information on overall obesity, while waist circumference and waist-to-hip ratio are more indicative of central obesity, which has an even stronger association with adverse metabolic changes in the body. However, research comparing the predictive power of BMI and waist measurements for diabetes has yielded conflicting results^22,23^. It appears, however, that using a combination of these measurements is a robust predictor and preferable to using them individually^24^.

Although type 2 diabetes has a strong genetic basis, this study has primarily focused on phenotypical features. While multiple genetic loci have been associated with a higher risk of type 2 diabetes, their ability to accurately predict the onset of the disease has been shown to be, at best, modest^25,26^. When incorporated into models that already include established risk factors and family history of diabetes, the improvement in precision is minimal or non-existent^27^. In our study, we found that family history of diabetes was a notable predictor for diabetes. Previous research has indicated that having knowledge of first-degree relatives with diabetes is a more robust predictor than established genetic variants for type 2 diabetes^27^. This suggests that family history captures not only the entire heritable genetic information, including unidentified risk genes, but also non-genetic factors such as behaviors and habits. At present, our understanding of the heritability of type 2 diabetes does not support the inclusion of genetic risk factors in prediction models. However, this may change in the future as our knowledge of the complex genetics underlying the disease expands.

Sex differences in important predictors of diabetes were observed in our study. For males, two markers associated with kidney function (microalbumin in urine and cystatin C) were among the top 10 predictors. Interestingly, our findings demonstrated that urate is one of the most significant predictors of type 2 diabetes, surpassing well-established factors like activity level and dietary habits. Notably, the predictive power of urate was stronger for women than men. This observation is consistent with previous studies, including a study in China which found that high urate levels were associated with an increased risk of diabetes only in women, but not in men^28^.

Imbalanced data is a frequent obstacle when developing classification models for conditions like type 2 diabetes. Given that a majority of participants will not develop the disease, the data leans heavily towards the larger class (no diabetes), yielding a diminutive minority class (diabetes). This disproportion often biases model development, with many algorithms giving precedence to correctly identifying the majority class. To address this, our study employed downsampling of the majority class during model training.

Despite these measures, when examining evaluation metrics sensitive to data imbalance, it's evident that our model doesn't consistently hit the mark. Research indicates that compact, clean datasets with a limited number of samples and features tend to produce more accurate predictions^7^. However, in alignment with our primary objective, we chose to retain as much data as feasible, acknowledging that this might compromise predictive performance. It's pivotal to emphasize that the intent of this model isn't to predict but strictly to identify and prioritize feature significance.

Another limitation of the study include the "healthy volunteer" effect, where participants in UK Biobank tend to be healthier than the general population^29^. Additionally, since the cohort mostly consists of middle-aged, mostly white individuals residing in the UK, the generalizability of the results may be limited to similar populations. A further constraint is the absence of C-peptide and antibodies in the UK Biobank dataset. Therefore, some individuals may be misclassified as having developed type 2 diabetes when they have developed type 1 diabetes or LADA (Latent autoimmune diabetes in adults). Furthermore, C-peptide could potentially serve as an important predictor since it is a measure of insulin production.

Our predictive model includes pre-diabetic individuals, which aligns with its intended application across a broad non-diabetic population. While this inclusion may elevate certain metrics, such as the ROC-AUC, it also ensures the model's clinical utility in identifying those at the highest risk who could derive significant benefit from early intervention strategies. A sensitivity analysis, provided in the supplementary material, demonstrates consistent model performance even when pre-diabetics are excluded, underscoring the model's stability. We acknowledge the potential impact on the significance of predictors as a limitation and suggest avenues for further research to enhance the model's precision.

This study has a significant strength in its extensive and largely unselected dataset, which allows for an unbiased analysis. To our knowledge, it is the most comprehensive study on phenotypical factors to predict future diabetes. Additionally, the study employs state-of-the-art machine learning algorithms such as XGboost and Shap Values, which adds to the robustness and accuracy of the results.

## Conclusion

The results of this study suggest that easily measurable biological factors are the most significant predictors of type 2 diabetes, outperforming known risk factors such as dietary factors, physical activity level, and socioeconomic status. The study also demonstrates that high accuracy in predicting type 2 diabetes can be achieved using only the 10 most important features, while the addition of numerous other factors only marginally improving precision.

### Supplementary Information

Supplementary Information.

## Data Availability

The datasets used and analyzed in this study originate from the UK Biobank under project ID 70236. Since the datasets are the property of UK Biobank, they are not available for direct request. However, interested researchers can apply for access through the UK Biobank Access Management System at https://www.ukbiobank.ac.uk/enable-your-research/access-our-data/.

## Abbreviations

GGT: Gamma-glutamyl transferase
BMI: Body mass index
ROC-AUC: Receiver operating characteristic area under the curve
eGFR: Estimated glomerular filtration rate
