# Supplementary material for: Identifying top ten predictors of type 2 diabetes through machine learning analysis of UK Biobank data
Source: Sci Rep. 2024 Jan 24;14:2102. doi: 10.1038/s41598-024-52023-5 (PMC10808323; doi:10.1038/s41598-024-52023-5)
Supplement: Supplementary file 1 — Supplementary Information. [file 41598_2024_52023_MOESM1_ESM.pdf]

Supplemental Material

Identifying Top Ten Predictors of Type 2 Diabetes Through

Machine Learning Analysis of UK Biobank Data

Moa Lugner[1*], Araz Rawshani[1], Edvin Helleryd[1], Björn Eliasson[1,2]

[1] University of Gothenburg, Sahlgrenska academy, Institute of Medicine, Sweden

[2] Sahlgrenska University Hospital, Department of Medicine, Gothenburg, Sweden

Correspondence: Moa.lugner@gu.se

Table of Contents

List of variables included in the model *4*

Touchscreen questionnaire or verbal interview 4

Medication 7

Physical measures 8

Biochemistry 9

Medical conditions 11

Female-specific factors: 13

Male-specific factors: 13

Shap summary graph for reduced model *15*

Predictors for the reduced model 15

Shap summary graph for sex specific models *16*

Predictors for sex-specific models 16

Top 50 predictors for main model *17*

Performance metrics for model excluding pre-diabetic subjects *18*

List of variables included in the model

Touchscreen questionnaire or verbal interview

Age at recruitment
Length of time at current address
Number in household
Number of vehicles in household
Average total household income before tax
Distance between home and job workplace
Time employed in main current job
Length of working week for main job
Frequency of travelling from home to job workplace
Job involves mainly walking or standing
Job involves heavy manual or physical work
Qualifications
Age completed full time education
Sex
Type of accommodation lived in
Own or rent accommodation lived in
Gas or solid-fuel cooking/heating
How are people in household related to participant
Current employment status
Transport type for commuting to job workplace
Job involves shift work
Ethnic background
Attendance/disability/mobility allowance
Private healthcare
IPAQ activity group
MET minutes per week for moderate activity
MET minutes per week for vigorous activity
MET minutes per week for walking
Summed days activity
Summed minutes activity
Length of mobile phone use
Weekly usage of mobile phone in last 3 months
Hands-free device/speakerphone use with mobile phone in last 3 month
Plays computer games
Sleep duration
Getting up in morning
Morning/evening person (chronotype)
Nap during day
Sleeplessness / insomnia
Daytime dozing / sleeping (narcolepsy)
Past tobacco smoking
Exposure to tobacco smoke at home
Exposure to tobacco smoke outside home
Time spend outdoors in summer

Time spent outdoors in winter
Skin colour
Ease of skin tanning
Childhood sunburn occasions
Use of sun/uv protection
Frequency of solarium/sunlamp use
Age first had sexual intercourse
Lifetime number of sexual partners
relative_diabetes_score:
Above moderate/vigorous recommendation
Above moderate/vigorous/walking recommendation
Difference in mobile phone use compared to two years previously
Usual side of head for mobile phone use
Snoring
Ever smoked
Smoking status
Current tobacco smoking
Smoking/smokers in household
Hair colour (natural, before greying)
Facial ageing
Ever had same-sex intercourse
Breastfed as a baby
Comparative body size at age 10
Comparative height size at age 10
Handedness (chirality/laterality)
Adopted as a child
Part of a multiple birth
Maternal smoking around birth
Alcohol intake frequency
Average weekly red wine intake
Average weekly champagne plus white wine intake
Average weekly beer plus cider intake
Average weekly spirits intake
Average weekly fortified wine intake
Alcohol intake versus 10 years previously
total_number_of_units_weekly
raw_and_cooked_veg
fresh_and_dried_fruit
Oily fish intake
Non-oily fish intake
Processed meat intake
Poultry intake
Beef intake
Lamb/mutton intake
Pork intake
Cheese intake
Bread intake

Cereal intake
Salt added to food
Tea intake
Hot drink temperature
Water intake
Variation in diet
Alcohol drinker status
Alcohol usually taken with meals
Reason for reducing amount of alcohol drunk
Never eat eggs, dairy, wheat, sugar
Milk type used
Spread type
Non-butter spread type details
Bread type
Cereal type
Coffee type
Major dietary changes in the last 5 years
Frequency of friend/family visits
Able to confide
Neuroticism score
Frequency of depressed mood in last 2 weeks
Frequency of unenthusiasm / disinterest in last 2 weeks
Frequency of tenseness / restlessness in last 2 weeks
Frequency of tiredness / lethargy in last 2 weeks
Leisure/social activities
Mood swings
Miserableness
Irritability
Sensitivity / hurt feelings
Fed-up feelings
Nervous feelings
Worrier / anxious feelings
Tense / 'highly strung'
Worry too long after embarrassment
Suffer from 'nerves'
Loneliness, isolation
Guilty feelings
Risk taking
Seen doctor (GP) for nerves, anxiety, tension or depression
Seen a psychiatrist for nerves, anxiety, tension or depression
Ever depressed for a whole week
Ever unenthusiastic/disinterested for a whole week
Ever manic/hyper for 2 days
Ever highly irritable/argumentative for 2 days
Illness, injury, bereavement, stress in last 2 years
Overall health rating
Falls in the last year

Long-standing illness, disability or infirmity
Weight change compared with 1 year ago
Wheeze or whistling in the chest in last year
Shortness of breath walking on level ground
Leg pain on walking
Pain type(s) experienced in last month
Chest pain or discomfort

Medication

N02 - Analgesics
A01 - Stomatological preparations
B01 - Antithrombotic agents
C01 - Cardiac therapy
G02 - Other gynecologicals
M01 - Anti-inflammatory and antirheumatic products
M02 - Topical products for joint and muscular pain
R02 - Throat preparations
C10 - Lipid modifying agents
A02 - Drugs for acid-related disorders
A11 - Vitamins
C03 - Diuretics
C09 - Agents acting on the renin-angiotensin system
C08 - Calcium channel blockers
H03 - Thyroid therapy
C07 - Beta blocking agents
R03 - Drugs for obstructive airway diseases
A10 - Drugs used in diabetes
D11 - Other dermatological preparations
S01 - Ophthalmologicals
N06 - Psychoanaleptics
M05 - Drugs for treatment of bone diseases
C02 - Antihypertensives
R06 - Antihistamines for systemic use
M04 - Antigout preparations
A12 - Mineral supplements
G04 - Urologicals
A07 - Antidiarrheals, intestinal anti-inflammatory/anti-infective agents
D07 - Corticosteroids, dermatological preparations
R01 - Nasal preparations
B03 - Antianemic preparations
P01 - Antiprotozoals
C05 - Vasoprotectives
H02 - Corticosteroids for systemic use
S02 - Otologicals
S03 - Ophthalmological and otological preparations
L01 - Antineoplastic agents
L04 - Immunosuppressants
R05 - Cough and cold preparations

A03 - Drugs for functional gastrointestinal disorders
N03 – Antiepileptics
G03 - Sex hormones and modulators of the genital system
N05 - Psycholeptics
L02 - Endocrine therapy
A06 - Laxatives
N07 - Other nervous system drugs
D06 - Antibiotics and chemotherapeutics for dermatological use
G01 - Gynecological antiinfectives and antiseptics
J01 - Systemic antibacterials
D01 - Antifungals for dermatological use
D02 - Emollients and protectives
B02 - Antihemorrhagics
D05 - Antipsoriatics
A08 - Antiobesity preparations, excluding diet products
M03 - Muscle relaxants
V03 - All other therapeutic products
J05 - Antivirals for systemic use
V06 - General nutrients
D10 - Anti-acne preparations
N04 - Anti-Parkinson drugs
A09 - Digestives, including enzymes
B05 - Blood substitutes and perfusion solutions
J07 - Vaccines
A05 - Bile and liver therapy
D08 - Antiseptics and disinfectants
H01 - Pituitary and hypothalamic hormones and analogues
N01 - Anesthetics
D03 - Preparations for treatment of wounds and ulcers
M09 - Other drugs for disorders of the musculo-skeletal systemV04
D04 - Antipruritics, including antihistamines, anesthetics, etc.
D09 - Medicated dressings
J02 - Antimycotics for systemic use
J04 - Drugs for tuberculosis
C04 - Peripheral vasodilators
A04 - Antiemetics and antinauseants
P03 - Ectoparasiticides, including scabicides
L03 - Immunostimulants
A16 - Other alimentary tract and metabolism products
H04 - Pancreatic hormones
H05 - Calcium homeostasis

Physical measures

diastolic_bp_mean
systolic_bp_mean
pulse_pressure
mean_arterial_pressure
Pulse wave Arterial Stiffness index

Hand strength
Waist circumference
Weight
Body mass index (BMI)
Hip circumference
Standing height
Sitting height
waist_hip_ratio
Leg fat-free mass (right)
Leg predicted mass (left)
Leg predicted mass (right)
Arm fat percentage (left)
Arm fat percentage (right)
Arm fat mass (left)
Arm fat mass (right)
Arm fat-free mass (right)
Arm fat-free mass (left)
Arm predicted mass (left)
Arm predicted mass (right)
Trunk fat percentage
Trunk fat mass
Trunk fat-free mass
Trunk predicted mass
Body fat percentage
Whole body fat mass
Whole body fat-free mass
Whole body water mass
Leg fat percentage (left)
Leg fat percentage (right)
Leg fat mass (left)
Leg fat mass (right)
Leg fat-free mass (left)
Impedance of whole body
Impedance of arm (left)
Impedance of arm (right)
Impedance of leg (left)
Impedance of leg (right)
bone_density_mean
bone_density_mean_tscore
Forced expiratory volume in 1-second (FEV1), Best measure
Forced vital capacity (FVC), Best measure
Egfr

Biochemistry

Alanine aminotransferase
Albumin
Alkaline phosphatase

Apolipoprotein A
Apolipoprotein B
Aspartate aminotransferase
C-reactive protein
Calcium
Cholesterol
Creatinine
Cystatin C
Direct bilirubin
Gamma glutamyltransferase
Glucose
Glycated haemoglobin (HbA1c)
HDL cholesterol
IGF-1
LDL direct
Lipoprotein A
Phosphate
SHBG
Testosterone
Total bilirubin
Total protein
Triglycerides
Urate
Urea
Vitamin D
Basophill count
Basophill percentage
Eosinophill count
Eosinophill percentage
Haematocrit percentage
Haemoglobin concentration
High light scatter reticulocyte count
High light scatter reticulocyte percentage
Immature reticulocyte fraction
Lymphocyte count
Lymphocyte percentage
Mean corpuscular haemoglobin
Mean corpuscular haemoglobin concentration
Mean corpuscular volume
Mean platelet (thrombocyte) volume
Mean reticulocyte volume
Mean sphered cell volume
Monocyte count
Monocyte percentage
Neutrophill count
Neutrophill percentage
Nucleated red blood cell count

Nucleated red blood cell percentage
Platelet count
Platelet crit
Platelet distribution width
Red blood cell (erythrocyte) count
Red blood cell (erythrocyte) distribution width
Reticulocyte count
Reticulocyte percentage
White blood cell (leukocyte) count
Creatinine (enzymatic) in urine
Potassium in urine
Sodium in urine

Medical conditions

AB_Diagnosis
C_Diagnosis
D_to_4Diagnosis
D_5_to_8Diagnosis
EDiagnosis
E_2_3Diagnosis
E_4_5Diagnosis
E_6Diagnosis
E_7Diagnosis
G_Diagnosis
H_Diagnosis
I0_Diagnosis
I1_Diagnosis
I20_to_25_Diagnosis
I26_to_28_Diagnosis
I30_I31_I32_I40_I41_Diagnosis
I33_to_I39_Diagnosis
I42_I43_Diagnosis
I48_Diagnosis
I50_Diagnosis
I44_I45_I46_I47_I49_I51_I52_Diagnosis
I6_Diagnosis
I7_Diagnosis
I8_Diagnosis
I9_Diagnosis
J0_Diagnosis
J1_Diagnosis
J2_Diagnosis
J3_Diagnosis
J4_Diagnosis
J8_Diagnosis
J9_Diagnosis
K0_1_Diagnosis

K2_K30_K31_Diagnosis
K35_K36_K37_38_Diagnosis
K4_Diagnosis
K50_K51_K52
K55_to_K64
K65_K66_K67
K7_Diagnosis
K8_Diagnosis
K9_Diagnosis
L_Diagnosis
M1_M2_Diagnosis
M3_Diagnosis
M4_M5_Diagnosis
M6_M7_Diagnosis
M8_M9_Diagnosis
N0_Diagnosis
N10_to_N16_Diagnosis
N17_N18_N19_Diagnosis
N20_to_N23_Diagnosis
N25_to_N29_Diagnosis
N3_Diagnosis
N4_N5_Diagnosis
N6_Diagnosis
N7_Diagnosis
N8_Diagnosis
N9_Diagnosis
O0_Diagnosis
O1_Diagnosis
O2_Diagnosis
O3_O4_Diagnosis
O6_O7_Diagnosis
O80_to_O84_Diagnosis
O85_to_O92_Diagnosis
O94_to_O99_Diagnosis
Q_Diagnosis
R0_Diagnosis
R1_Diagnosis
R20_to_R23_Diagnosis
R25_to_R29_Diagnosis
R3_Diagnosis
R40_to_R46_Diagnosis
R47_to_R49_Diagnosis
R5_R6_Diagnosis
R7_Diagnosis
R80_to_R82_Diagnosis
R83_to_R89_Diagnosis
R90_to_R94_Diagnosis

S_Diagnosis
xx_Diagnosis

Female-specific factors:

Ever had breast cancer screening / mammogram
Years since last breast cancer screening / mammogram
Ever had cervical smear test
Years since last cervical smear test
Age when periods started (menarche)
Had menopause
Age at menopause (last menstrual period)
Time since last menstrual period
Length of menstrual cycle
Menstruating today
Number of live births
Birth weight of first child
Age of primiparous women at birth of child
Age at first live birth
Age at last live birth
Ever had stillbirth, spontaneous miscarriage or termination
Number of stillbirths
Number of spontaneous miscarriages
Number of pregnancy terminations
Ever taken oral contraceptive pill
Type of progestan-only oral contraceptive used (pilot)
Age started oral contraceptive pill
Age when last used oral contraceptive pill
Ever used hormone-replacement therapy (HRT)
Age started hormone-replacement therapy (HRT)
Age last used hormone-replacement therapy (HRT)
Ever had hysterectomy (womb removed)
Age at hysterectomy
Bilateral oophorectomy (both ovaries removed)
Age at bilateral oophorectomy (both ovaries removed)

Male-specific factors:

Relative age of first facial hair
Relative age voice broke
Hair/balding pattern
Number of children fathered

Variables Created for Analyses:

relative_diabetes_score:
This score quantifies the presence of diabetes in first-degree relatives of the individual. The score is derived from three fields: “illness of father”, “illness of mother”, and “illness of sibling”. A score of 0 indicates that none of the first-degree relatives (father, mother, or

sibling) have diabetes. A score of 1 indicates that one of the first-degree relatives has diabetes.A score of 2 denotes that two or more first-degree relatives have diabetes.

total_number_of_units_weekly:
This variable represents the estimated weekly consumption of alcohol units by participants. The derivation process is as follows:

In the UK Biobank study, participants provided their estimated consumption by specifying the number of glasses of different alcoholic beverages they drink per month.
Each type of drink was converted into units of alcohol based on its alcohol content. For reference, one unit corresponds to 10ml or 8g of pure alcohol.
To transform the monthly consumption estimation into a weekly format, the total monthly units for each participant were divided by 4.345.
This calculation provides an approximate weekly consumption of alcohol units for each individual.

pulse_pressure:
This variable is derived from the difference between systolic and diastolic blood pressure measurements. The formula used is:
pulse pressure = systolic blood pressure (mean) − diastolic blood pressure (mean)

mean_arterial_pressure:
This variable is an estimate of the average arterial pressure during a single cardiac cycle. It is calculated using the formula: MAP = diastolic blood pressure (mean) + 1/3(pulse_pressure)

Egfr
Egfr was calculated using the function CKDEpi.creat in the "nephro-package in R. It utilizes creatinine, sex, age and ethnicity to calculate egfr using the established CKD-EPI equation.

Shap summary graph for reduced model

Predictors for the reduced model

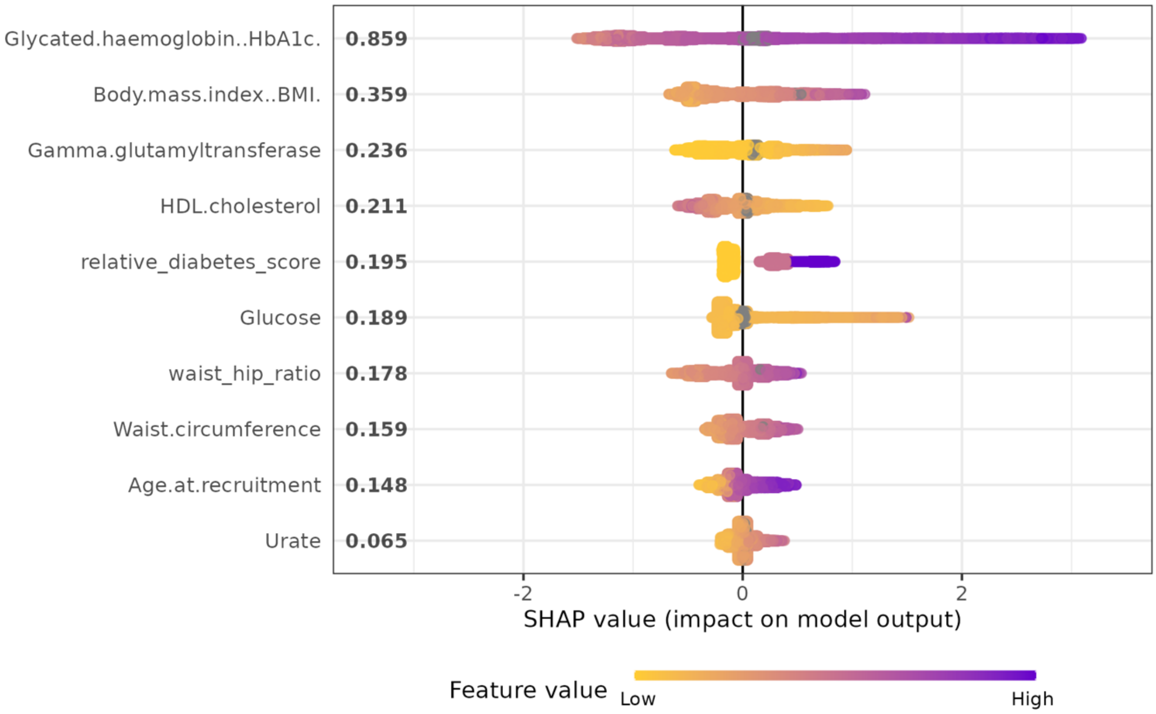

Shap summary for the reduced model

Shap summary graph for sex specific models

Predictors for sex-specific models

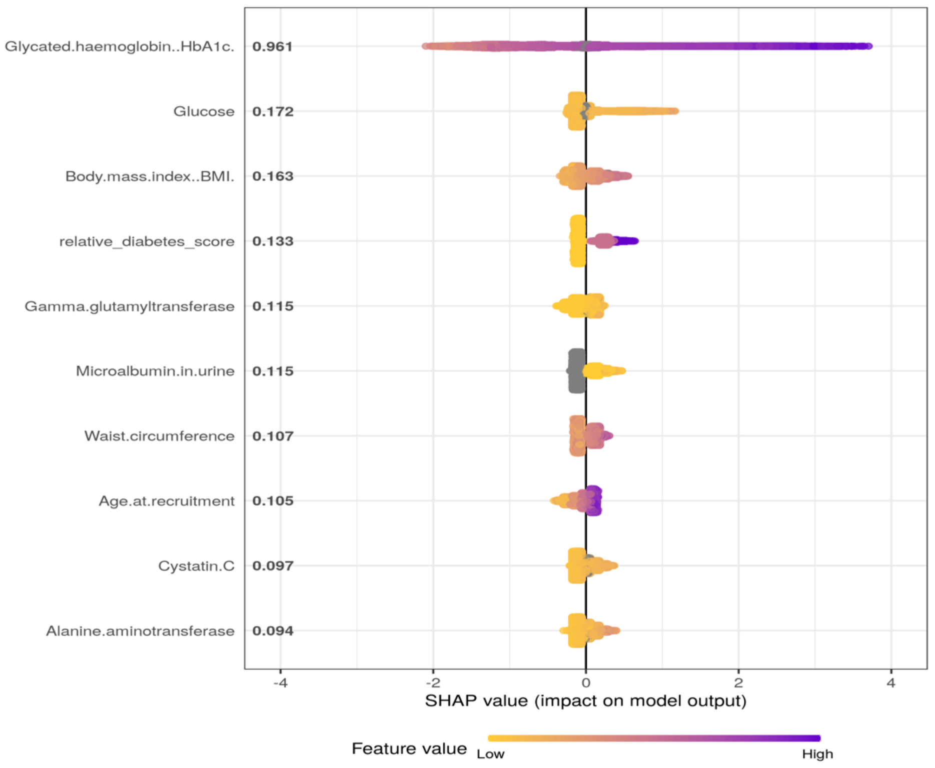

Shap summary graph for male cohort

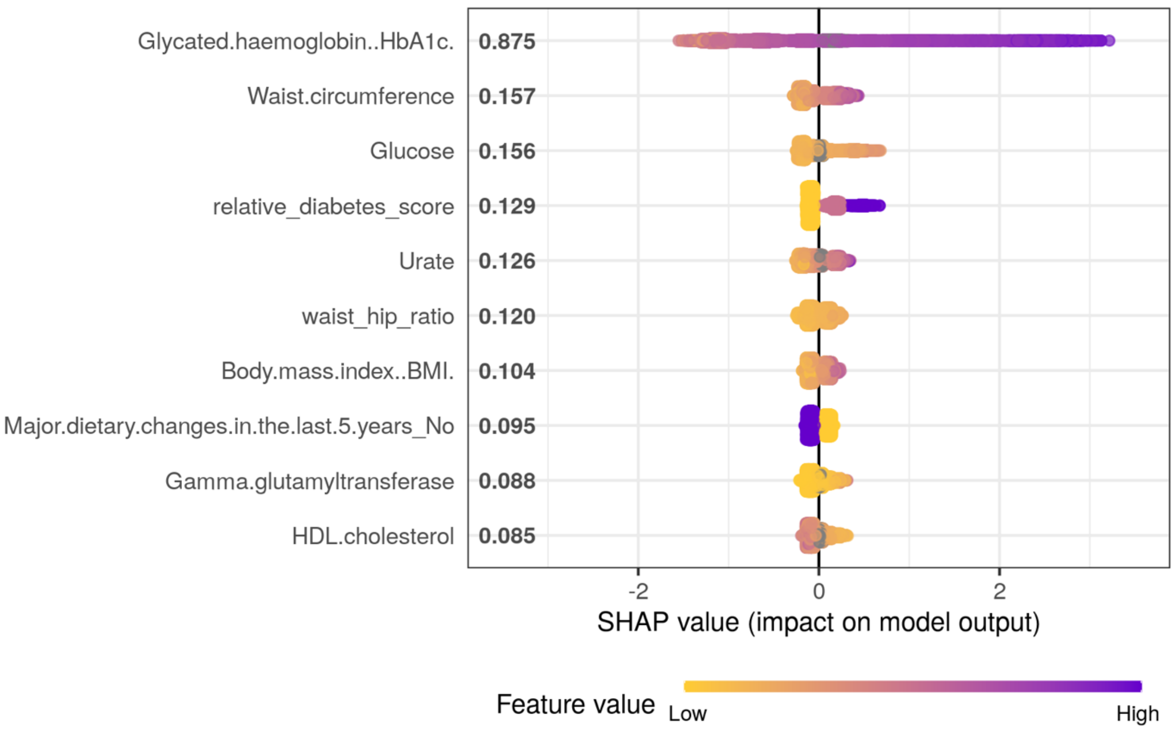

Shap summary graph for female cohort

Top 50 predictors for main model

Top 50 Features Based on SHAP Values	
Feature	Mean SHAP Value
Hba1C	0.95
BMI	0.19
Waist circumference	0.19
Glucose	0.16
relative_diabetes_score	0.16
Gamma-glutamyltransferase	0.12
Waist-hip ratio	0.12
HDL cholesterol	0.09
Age at recruitment	0.08
Urate	0.08
Alanine aminotransferase	0.07
Weight change compared with 1 year ago lost weight	0.07
Vitamin D	0.07
Aspartate aminotransferase	0.07
Reticulocyte count	0.07
Haemoglobin concentration	0.06
Sodium in urine	0.06
Longstanding illness, disability or infirmity No	0.06
Cystatin C	0.05
Weekly alcohol intake	0.05
Triglycerides	0.05
A02 Drugs for acid-related disorders	0.05
SHBG	0.05
Longstanding illness, disability or infirmity _Yes	0.05
N02 Analgesics	0.05
Pulse pressure	0.04
Major dietary changes. In the last 5 years_No	0.04
Creatinine	0.04
Mean sphered cell volume	0.04
Reticulocyte percentage	0.04
Grip strength	0.04
Platelet distribution width	0.04
Platelet crit	0.04
Attendance, disability, mobility, allowance_None.of.the.above	0.04
Comparative body size at age 10_Thinner	0.04
Systolic blood pressure	0.04
Red blood cell erythrocyte distribution width	0.03
Urea	0.03
Mean corpuscular haemoglobin	0.03
Potassium in urine	0.03
Overall health rating_Fair	0.03
Direct bilirubin	0.03
Average weekly spirits intake	0.03
Cholesterol	0.03
Lymphocyte count	0.03
IGF.1	0.03
C09 Agents acting on the renin-angiotensin system	0.03

Forced expiratory volume in 1 second FEV1...Best.measure	0.03
Daytime dozing, sleeping, narcolepsy._Never.rarely	0.03
K55_to_K64	0.03

Performance metrics for model excluding pre-diabetic subjects

accuracy	binary	0.9233028
precision	binary	0.1736723
recall	binary	0.6081551
f_meas	binary	0.2701867
roc_auc	binary	0.8902863

Table with performance metrics of additional model where all participants with 6.1 or higher p-glucose at baseline were excluded.
